# Comparative analysis demonstrates cell type-specific conservation of SOX9 targets between mouse and chicken

**DOI:** 10.1038/s41598-019-48979-4

**Published:** 2019-08-29

**Authors:** Satoshi Yamashita, Kensuke Kataoka, Hiroto Yamamoto, Tomoko Kato, Satoshi Hara, Katsushi Yamaguchi, Claire Renard-Guillet, Yuki Katou, Katsuhiko Shirahige, Haruki Ochi, Hajime Ogino, Tokujiro Uchida, Masafumi Inui, Shuji Takada, Shuji Shigenobu, Hiroshi Asahara

**Affiliations:** 1 Department of Systems BioMedicine, Tokyo Medical and Dental University, 1-5-45 Yushima, Bunkyo-ku, Tokyo 113-8510 Japan; 2 Department of Systems BioMedicine, National Institute for Child Health and Development, 2-10-1 Okura, Setagaya-ku, Tokyo 157-8535 Japan; 3 Research Fellow of Japan Society for the Promotion of Science, Tokyo, Japan; 4 Department of Anesthesiology, Tokyo Medical and Dental University, Graduate School of Medical and Dental Sciences, 1-5-45 Yushima, Bunkyo-ku, Tokyo 113-8510 Japan; 5 Functional Genomics Facility, National Institute for Basic Biology, 38, Nishigonaka Myodaiji, Okazaki, Aichi 444-8585 Japan; 6 Laboratory of Genome Structure and Function Center for Epigenetic Disease, Institute of Molecular and Cellular Biosciences, The University of Tokyo, 1-1-1 Yayoi, Bunkyo-ku, Tokyo 113-0032 Japan; 7 Institute for Promotion of Medical Science Research, Faculty of Medicine, Yamagata University, 2-2-2 Iida-nishi, Yamagata, 990-9585 Japan; 8 Amphibian Research Center, Hiroshima University, 1-3-1 Kagami-yama, Higashi-Hiroshima, Hiroshima 739-8526 Japan; 9 Laboratory of Animal Regeneration Systemology, Department of Life Sciences, School of Agriculture, Meiji University, 1-1-1 Higashi-Mita Tama-ku, Kawasaki, Kanagawa 214-8571 Japan; 10 Department of Molecular Medicine, The Scripps Research Institute, California, 92037 USA; 11 AMED-CREST, Japan Agency for Medical Research and Development (AMED), Tokyo, Japan

**Keywords:** Gene regulation, Transcription

## Abstract

SRY (sex-determining region Y)-box 9 (SOX9) is a transcription factor regulating both chondrogenesis and sex determination. Among vertebrates, SOX9’s functions in chondrogenesis are well conserved, while they vary in sex determination. To investigate the conservation of SOX9’s regulatory functions in chondrogenesis and gonad development among species, we performed chromatin immunoprecipitation sequencing (ChIP-seq) using developing limb buds and male gonads from embryos of two vertebrates, mouse and chicken. In both mouse and chicken, SOX9 bound to intronic and distal regions of genes more frequently in limb buds than in male gonads, while SOX9 bound to the proximal upstream regions of genes more frequently in male gonads than in limb buds. In both species, SOX palindromic repeats were identified more frequently in SOX9 binding regions in limb bud genes compared with those in male gonad genes. The conservation of SOX9 binding regions was significantly higher in limb bud genes. In addition, we combined RNA expression analysis (RNA sequencing) with the ChIP-seq results at the same stage in developing chondrocytes and Sertoli cells and determined SOX9 target genes in these cells of the two species and disclosed that SOX9 targets showed high similarity of targets in chondrocytes, but not in Sertoli cells.

## Introduction

SRY (sex-determining region Y)-box 9 (SOX9) is a transcription factor containing a high mobility group (HMG) box DNA binding domain, which recognizes the (A/T)(A/T)CAA(T/A)G DNA sequence and controls the expressions of target genes^1–3^. A *SOX9* mutation causes campomelic dysplasia (CD), which is characterized by skeletal deformity and sex reversal of XY patients^4,5^. Analysis of genetically modified *Sox9* heterozygous mutations in mice revealed skeletal deformation similar to that of patients with CD^6^. Moreover, the homozygous mutation is lethal at the embryonic stage^7^. *Sox9* inactivation in limb buds using the Cre/*loxP* recombination system revealed that SOX9 has an essential function on mesenchymal condensation and subsequent cartilage formation^8^. By contrast, ectopic expression of *Sox9* in XX mice gonads induced testis formation, while XY gonad-specific inactivation of *Sox9* resulted in the absence of testes^9,10^. These data clearly indicated that SOX9 is a critical regulator of chondrogenesis and sex determination.

The structure and expression of *SOX9* in chondrocytes are well conserved among vertebrates^11,12^. In chondrogenesis, SOX9 directly promotes the expression of chondrocyte-specific extracellular matrix genes, including *COL2A1* (encoding collagen type II alpha 1 chain) and *COL11A2* (encoding collagen type XI alpha 2 chain), which are critical for early chondrogenesis^13–15^. *col2a1* regulation by Sox9 was also demonstrated in zebrafish cartilage development^15,16^, indicating evolutionary conservation of target genes. These observations suggested evolutionary conservation of transcriptional networks as well as the function of SOX9 in chondrogenesis.

In contrast, while *SOX9* expression is upregulated by the male sex determinant, SRY, and SOX9 plays a central role in determining mammalian male sex by promoting Sertoli cell differentiation^17,18^, *sox9* is not expressed during testis development in *Xenopus tropicalis*^19^ and the *SOX9* ortholog *sox9b* knockout mutants showed female-to-male sex reversal in medaka (*Oryzias latipes*)^20^. In addition, although the *SOX9* gene is expressed in the developing testis in mouse and chicken, *Amh* (encoding anti-Müllerian hormone), which is involved in Müllerian duct regression, is expressed after *Sox9* expression in mouse gonad, whereas *AMH* expression precedes *SOX9* in chicken gonads^21,22^. These findings suggested that the regulatory function of SOX9 in gonad development is not strictly conserved among vertebrates.

Recently, studies using chromatin immunoprecipitation sequencing (ChIP-seq) have precisely determined the functions of SOX9 in chondrogenesis, mainly in mice^23,24^. In addition, another ChIP-seq study using male gonads of mouse and cattle showed that SOX9 could bind to and regulate a similar set of genes in these two mammals^25^. However, there has been no comprehensive analysis of the regulatory network and function of SOX9 between mammals and other vertebrates. Therefore, to gain insights into the conservation of SOX9’s regulatory functions in chondrogenesis and gonad development among distant vertebrates, we performed ChIP-seq using developing limb buds and male gonads from embryos of mouse and chicken. Chicken was chosen as a representative for comparison against mouse because, although chickens are vertebrates, they are not phylogenetically close to mice and are not mammals. The genome-wide comparative analysis showed similar characteristics of SOX9 binding regions, such as their positions within genes and their binding motifs. Furthermore, we combined RNA expression analysis (RNA sequencing (RNA-seq)) of chondrocytes and Sertoli cells at the same developmental stage with the ChIP-seq results and analyzed the presumed SOX9 target genes in these cells of the two species. We observed high similarity of SOX9 target genes in chondrocytes, but low similarity in Sertoli cells, indicating that the regulatory targets of SOX9 in testis development differ between the two species. The present study provided new insights into cell type-specific binding preferences of SOX9 and their conservation in chondrocytes and Sertoli cells between mouse and chicken.

## Results

### Identification of cell type-specific SOX9 binding regions in chondrocytes and Sertoli cells

**Figure 1:**
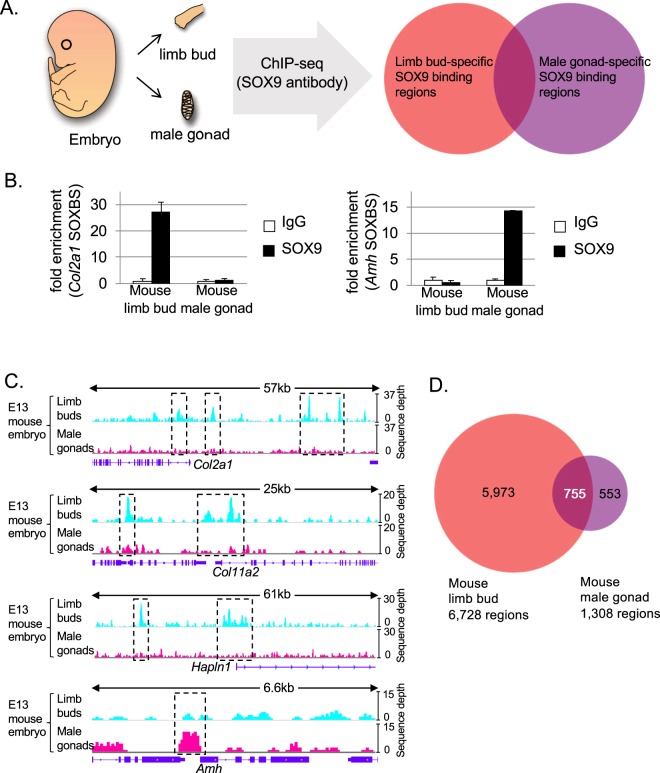
SOX9 binds to cell-specific DNA elements. (**A)** Schematic illustration of the tissue-specific SOX9 target identification strategy. **(B)** Chromatin immunoprecipitated SOX9 binding non-enriched (normal rabbit IgG) or enriched (SOX9) DNA in E13 mouse limb buds (Left) and male gonads (Right) was analyzed quantitatively using real-time PCR with site-specific primer sets for the *Col2a1* SOX9 binding site (SOXBS) and the *Amh* SOXBS, respectively. The fold-enrichment for SOX9-enriched DNA versus non-enriched DNA is shown. Mean values ± SD (three technical replicates) are shown. **(C)** Sequence tag accumulation of SOX9 Chromatin immunoprecipitation sequencing (ChIP-seq) of embryonic day 13 (E13) mouse limb buds and male gonads around *Col2a1*, *Col11a2*, *Hapln1*, and *Amh* are shown. **(D)** The number of SOX9 ChIP-seq peak regions and those that overlap between E13 mouse limb buds and male gonads are displayed in a Venn diagram. SOX9 ChIP-seq peak regions were determined by calculating ChIP-seq data using the MACS algorithm (p-value < 1 e-7 and false discovery rate (FDR) <1%). SOX9, SRY (sex-determining region Y)-box 9; Col2a1, collagen type II alpha 1 chain; Amh, anti-Müllerian hormone; Col11a2, collagen type XI alpha 2 chain; Hapln1, hyaluronan and proteoglycan link protein 1.

To identify the genomic locations of SOX9 binding regions in developing chondrocytes and Sertoli cells, we applied ChIP followed by deep sequencing (ChIP-seq) to developing limb buds and male gonads collected from embryonic day (E) 13 mice **(**Fig. 1A**)**. Before performing deep sequencing, we used quantitative PCR to examine whether SOX9 could bind to known binding regions of the chondrocyte-specific target *Col2a1*^13^ and the Sertoli cell-specific target *Amh*^26,27^ in each tissue. As expected, we detected SOX9 binding to the *Col2a1* enhancer in limb buds and to the *Amh* enhancer in male gonads **(**Fig. 1B**)**. This result prompted us to conduct deep sequencing with these samples. We observed sequence tag accumulation on known SOX9 binding regions of chondrocyte target genes, *Col2a1*, *Col11a2*, and *Hapln1* (encoding hyaluronan and proteoglycan link protein 1)^13,14,28^, in mouse E13 limb buds library and on the Sertoli cell target gene *Amh*^26,27^ in the male gonad library **(**Fig. 1C**)**, indicating the successful detection of SOX9 binding regions in the ChIP-seq experiment. Interestingly, SOX9 ChIP-seq peaks adjacent to *Amh* and those adjacent to *Col2a1*, *Col11a2*, and *Hapln1* were unique to the male gonad and limb bud, respectively, suggesting that SOX9 has a cell type differential binding profile. From this experiment, we identified 6,728 significantly enriched ChIP-seq peak regions bound by SOX9 in E13 limb buds and 1,308 regions in E13 male gonads, among which 755 regions overlapped between the two tissues **(**Fig. 1D, Supplementary Tables S1 and S2**)**.

### Comparative analysis of SOX9 binding regions demonstrate cell type-specific binding

**Figure 2:**
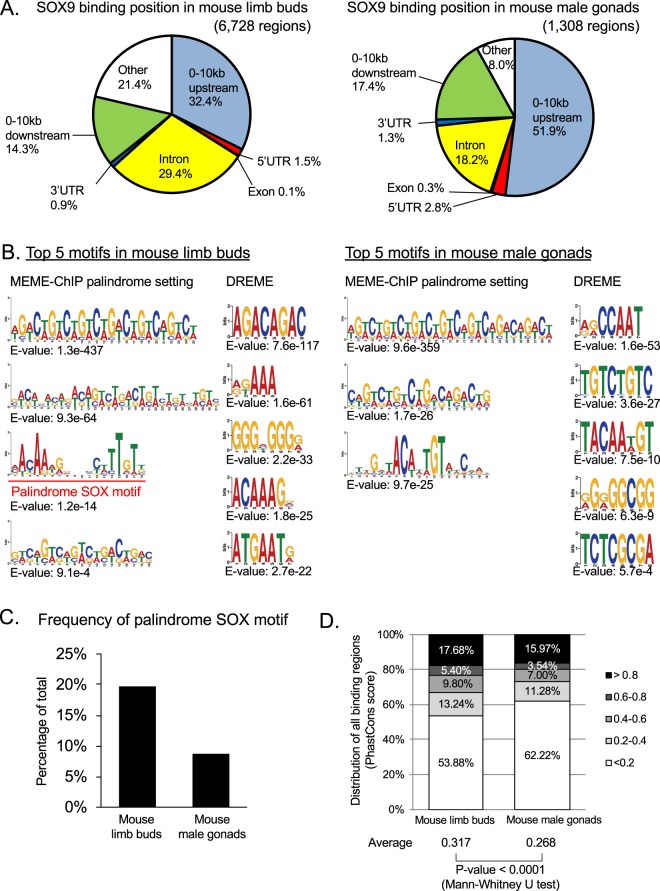
Comparative analysis indicates a cell type-specific binding of SOX9 and conservation state of SOX9 binding regions. **(A)** Genomic positions of SOX9 binding regions in embryonic day 13 (E13) mouse limb buds (Left) and in male gonads (Right) are displayed as pie charts. The genome is separated into seven regions: Regions upstream of the transcription start site (TSS) of genes up to 10 kb, 5′-untranslated region (UTR), exon, intron, 3′-UTR, 10 kb downstream regions from the end of 3′-UTR, and others. **(B)** Top 5 enriched SOX9 binding motifs in E13 mouse limb buds (Left) and in male gonads (Right) are shown. All results are listed in Supplementary Figs S1 and S2. The proportion of nucleotide occurrence is displayed by the letter size, which was calculated using the MEME-ChIP program. Palindrome setting means using the option that detects only palindromic sequences. DREME is a program included in MEME-ChIP and is suitable to detect short motifs. **(C)** Palindrome SOX motifs displayed in Fig. 2B with a red line and character were searched for SOX9 binding regions in each tissue using profit in EMBOSS and their distribution is shown. **(D)** Evolutionary conservation across 60 vertebrate genomes of SOX9 binding regions was analyzed by calculating the average phastCons scores. The average phastCons score distributions of SOX9 binding regions and total average scores in each tissue are shown. SOX9, SRY (sex-determining region Y)-box 9.

The SOX9 binding regions (6,728 ChIP-seq peak regions in limb buds and 1,308 in male gonads) were classified into seven types in terms of their positional relationship with genes: 0–10 kb upstream of the gene, 5′-untranslated region (UTR), coding exon, intron, 3′-UTR, 0–10 kb downstream, and other. The classification revealed a remarkable difference in the SOX9 occupation patterns between limb buds and male gonads: 32.4% of total SOX9 ChIP-seq peaks in the limb bud samples were found in the upstream region of the genes, while 51.9% of peaks for the male gonad samples were found in this category **(**Fig. 2A**)**. In brief, SOX9 binding to upstream regions was more abundant in the E13 male gonads than in the limb buds. In contrast, SOX9 bound to introns and other regions (regions distant from any genes) more frequently in the limb bud samples than in the male gonad samples **(**Fig. 2A**)**.

We examined SOX9 DNA binding motifs based on the enriched DNA sequences of ChIP-seq peak regions using MEME-ChIP software^29^ including an optional setting that detects only palindromic motifs and DREME^30^, which is suitable to detect short motifs. Our *de novo* motif analysis identified motifs similar to consensus SOX motif ((A/T)(A/T)CAA(A/T)G) (E-value on DREME: 1.8 e-25 in limb buds and 7.5 e-10 in male gonads), as well as the CAGA repeat and its reverse sequence in SOX9 ChIP-seq peaks in both limb buds and male gonads **(**Figs 2B, S1 and S2**)**. It has been suggested that SOX9 homodimers bind to enhancer regions of chondrocyte target genes containing the SOX palindromic motif^31,32^. Consistent with this, inverted Sox9 binding motifs separated by 4 bp, representing a SOX palindromic motif^31,32^, were enriched only in the limb bud SOX9 ChIP-seq peaks (E-value: 1.2 e-14 in the palindrome setting and 6.5 e-25 in the normal setting) but not in the male gonad data **(**Figs 2B, S1 and S2**)**. As a motif that seems to have another meaning, “CCAAT” was listed (E-value on DREME: 4.8 e-17 in limb buds and 1.6 e-53 in male gonads.) **(**Figs 2B, S1 and S2**)**. We then examined the frequency of SOX palindromic motifs in SOX9 binding regions of each cell type using profit in EMBOSS^33^. Consistent with the results of MEME-ChIP analysis, we found that palindrome SOX motifs were observed more frequently in limb bud SOX9 binding regions (19.65%) than in male gonad SOX9 binding regions (8.72%) **(**Fig. 2C**)**.

Taken together, the results of the comparison of SOX9 binding regions in developing mouse limb buds and male gonads showed that SOX9 binding regions were observed more frequently in introns and regions distant from genes and there were more palindromic SOX motifs in the chondrocyte SOX9 binging regions. By contrast, SOX9 binding regions were observed more frequently in proximal upstream regions of genes and there were fewer palindromic SOX motifs in Sertoli cell SOX9 binding regions.

### Conservation patterns of SOX9 targets between developing chondrocytes and Sertoli cells among mice and chickens

Conservation of tissue-specific SOX9 binding regions among vertebrates was assessed by calculating the average phastCons score, ranging from 0 to 1, which was generated by a multiple alignment of 60 vertebrate genomes^34^ around the SOX9 ChIP-seq peaks. The results indicated that 17.68% and 15.97% of the total E13 limb bud and male gonad SOX9 ChIP-seq peaks, respectively, were associated with a high phastCons score (>0.8) **(**Fig. 2D**)**. Even at a moderate conservation level, 5.40% and 3.54% of the regions in limb buds and male gonads, respectively, were associated with average phastCons scores between 0.6 and 0.8 **(**Fig. 2D**)**. SOX9 binding regions in limb buds were highly conserved compared with those in male gonads, with a significant difference in the average score (0.317 in limb buds and 0.268 in male gonads, *p* < 0.0001 by the Mann–Whitney U test) **(**Fig. 2D**)**. From these results, we decided to explore the genome-wide SOX9 binding regions in other non-mammalian vertebrate animals and examine the conservation of SOX9 targets in chondrocytes and Sertoli cells.

**Figure 3:**
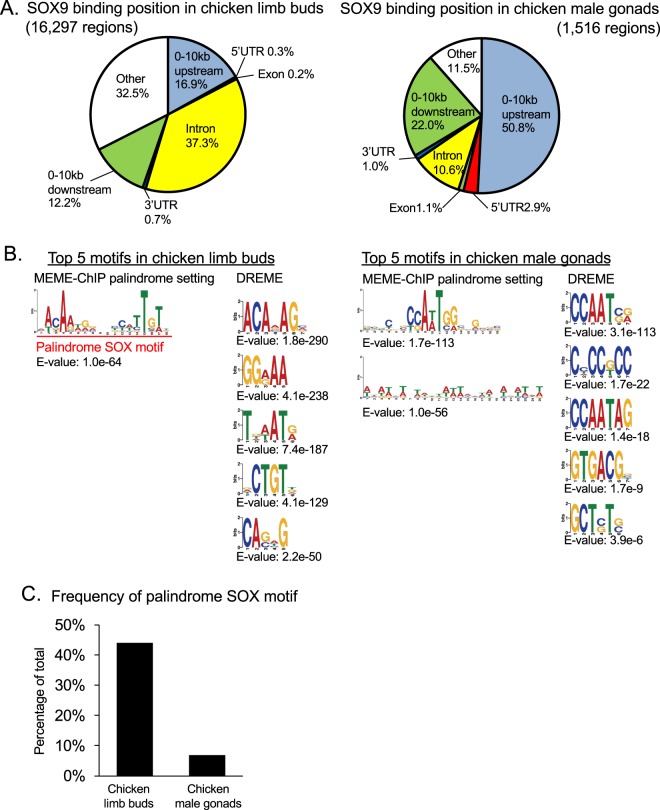
Identification of SOX9 binding regions and targets in developing chicken limb buds and male gonads. (**A)** Genomic positions of SOX9 binding regions in embryonic day 7 (E7) chicken limb buds (Left) and in male gonads (Right) are displayed in pie charts. Genomic partitions are the same as those used in Fig. 2A. **(B)** Top 5 enriched SOX9 binding motifs in E7 chicken limb buds (Left) and in male gonads (Right) are shown. All results are listed in Supplementary Figs S3 and S4. The representations are the same as in Fig. 2B. (**C**) Palindrome SOX motifs displayed in (**B**) with red line and characters were searched in the SOX9 binding regions in each tissue using profit in EMBOSS and their distributions are shown. SOX9, SRY (sex-determining region Y)-box 9.

Chicken was chosen as the representative of non-mammal vertebrates for comparison because its genomic database is suitable to conduct ChIP-seq analysis and abundant knowledge has been accumulated on chicken development, including chondrogenesis and gonad formation. Furthermore, E13 mice and HH30 (Hamburger-Hamilton stage 30^35^) chickens are known to be comparable stages in limb bud and gonad development^36,37^, which was also a reason for choosing chicken. We performed ChIP-seq analysis of chicken using comparable tissues at this developmental stage so that we could compare the ChIP-seq data directly between the two species. Our ChIP-seq analysis using E7 chicken embryonic limb buds and male gonads led to the identification of 16,297 and 1,516 significantly enriched SOX9 ChIP-seq peak regions, respectively **(**Supplementary Tables S3 and S4**)**. We analyzed the genomic position of the 16,297 SOX9 ChIP-seq peaks in chicken limb buds and 1,516 in male gonads and revealed that the positional relationship between the peaks and the genes had the same tendency as those in mice. That is, SOX9 binding to proximal upstream regions within 10 kb of genes was more common in E7 chicken male gonads and the binding to introns and regions distant from genes was more common in limb buds **(**Fig. 3A**)**. In addition, our *de novo* motif analysis in the chicken data identified palindromic SOX motif enrichment only in limb bud SOX9 binding regions (E-value: 1.0 e-64 in MEME-ChIP palindrome setting and 3.7 e-83 in MEME-ChIP normal setting) and reflecting this result, enrichment of single SOX motifs was also confirmed by DREME (E-value: 1.8 e-290 and 4.3 e-18) **(**Figs 3B and S3**)**. In male gonad SOX9 ChIP-seq peak regions, even single SOX motifs were not enriched and the CCAAT sequence was listed in our analysis (E-value: 3.1 e-113 in DREME and 6.1 e-325 in MEME-ChIP normal setting) **(**Figs 3B and S4**)**. We also examined the frequency of SOX palindromic motifs **(**Fig. 3B: Red line and character) in SOX9 binding regions of each chicken cell type and found that the palindromic SOX motif was observed more frequently in limb bud SOX9 binding regions (43.93%) than in male gonad SOX9 binding regions (6.79%) **(**Fig. 3C**)**. This observation that the palindrome SOX motif seems to have a function only in chondrocytes, was a common feature in mouse and chicken, and suggested that the cell type-specificity in chondrocytes and Sertoli cells is conserved between mouse and chicken.

**Figure 4:**
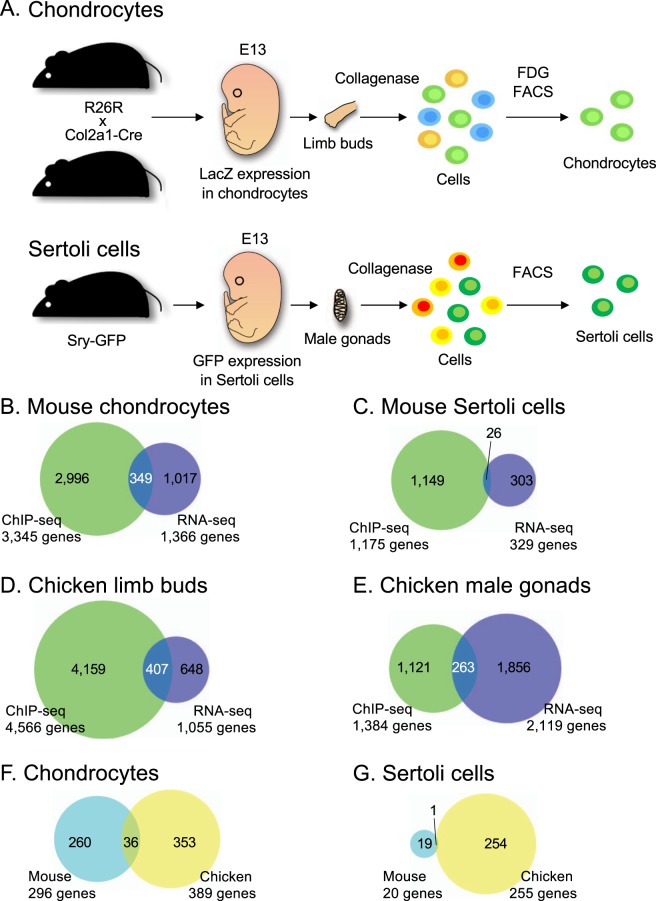
SOX9 targets in developing chondrocyte and Sertoli cells of mouse and chicken. (**A)** Schematic illustration of mouse chondrocyte preparation (upper) and mouse Sertoli cell preparation (lower) for RNA sequencing. Details are presented in the materials and methods section. R26R: Rosa 26 reporter mouse. Col2a1-Cre: *Col2a1*/Cre transgenic mouse. LacZ: β-D-galactosidase. FDG: fluorescein di-β-D-galactopyranoside. FACS: fluorescence activated cell sorting. Sry-GFP: *Sry*/GFP transgenic mouse. GFP: green fluorescent protein. **(B–E)** Venn diagrams of SOX9 target genes in mouse chondrocytes (**B**), mouse Sertoli cells (**C**), chicken limb buds (**D**), and chicken male gonads (**E**). The number of genes to which SOX9 binds in its cis-regulatory region in ChIP-seq and the number of genes highly expressed in RNA-seq are indicated. **(F,G)** Venn diagrams for the conservation of SOX9-regulated genes in chondrocytes (**F**) and Sertoli cells (**G**) are shown. The genes were compared after matching orthologs in an ortholog database. SOX9, SRY (sex-determining region Y)-box 9.

We then performed analyses to compare the SOX9 target genes in chondrocytes and Sertoli cells between mouse and chicken. We assigned a gene as a putative SOX9 target gene if the ChIP-seq peak corresponded to a cis-regulatory region (up to 10 kb upstream from the transcription start site (TSS)), 5′-UTR, exon, intron, or 3′-UTR of the gene. We identified 3,345 protein coding genes in mouse limb buds and 1,175 in mouse male gonads as putative SOX9 targets **(**Supplementary Tables S1 and S2**)**. Among these gene groups, to extract genes whose transcription is actually affected by SOX9, genes with high expression in each cell were examined using RNA sequencing. To obtain pure chondrocytes and pure Sertoli cells for RNA sequencing, we prepared β-D-galactosidase (lacZ)-labeled chondrocytes and green fluorescent protein (GFP)-labeled Sertoli cells using transgenic mice, and these cells were collected using fluorescence activated cell sorting (FACS) (see the Materials and Methods section and Fig. 4A). For each cell type, three biological replicate samples were prepared. Quartz-seq^38^, a highly sensitive RNA sequencing method, was chosen for the present experimental system because the numbers of obtained cells was small. To confirm the quality of the RNA-seq process, graphs of the PCA (principal component analysis) plot **(**Supplementary Fig. S5**)** were created and we judged that the variations among the replicated samples were small. Visualization of the RNA-seq reads showed consistent mapping patterns between replicates as shown in the regions around *COL2A1* and *COL9A3* (encoding collagen type IX alpha 3 chain) (cartilage related genes) and *AMH* and *DMRT1* (encoding doublesex and mab-3 related transcription factor 1) (Sertoli cell related genes) **(**Supplementary Fig. S6**)**. Thus, we judged that they were suitable samples for subsequent analysis. We defined a significantly highly expressed gene in chondrocytes and Sertoli cells when its expression level (as estimated using Fragment per kilobase of transcript per million fragments sequenced, FPKM) in each cell was more than 1.0, was 1.5 times higher than that in the whole embryo, and the q-value of the comparisons was smaller than 0.05 (FPKM >1.0, fold change of FPKM >1.5 and q-value < 0.05). We found that 1,366 genes **(**Supplementary Table S5**)** in mouse chondrocytes were significantly highly expressed according to this definition and 349 of them were included as putative SOX9 target genes from the ChIP-seq result **(**Fig. 4B**)**. We regarded these genes, showing relation with SOX9 in ChIP-seq and significantly high expression in RNA-seq, as SOX9-regulated genes in this study. In mouse Sertoli cells, 329 genes were significantly highly expressed according to the RNA sequencing results **(**Supplementary Table S6**)** and 26 of them were SOX9-regulated genes **(**Fig. 4C**)**. Among these tissue-specific SOX9-regulated genes, previously reported SOX9 target genes related to chondrogenesis, such as *COL2A1*, *COL9A1* (encoding collagen type IX alpha 1 chain), *COL9A2* (encoding collagen type IX alpha 2 chain), *COL11A2*, *COL27A1* (encoding collagen type XXVII alpha 1 chain), *ACAN* (encoding Aggrecan), *COMP* (encoding cartilage oligomeric matrix protein), and *HAPLN1*^7,13,14,28,39–43^ were listed in the E13 mouse chondrocyte data. In addition, SOX9 target genes related to gonad development, such as *Amh, Dhh* (encoding desert hedgehog), and *Nr5a1* (encoding nuclear receptor subfamily 5 group A member 1)^26,27,44^ were listed in the E13 mouse male gonad data, indicating the successful detection of SOX9 targets in each cell sample using our strategy.

Regarding chickens, it was impossible to use transgenic animals and prepare lacZ- or GFP-labeled cells; therefore, E7 chicken limb buds and male gonads were used as substitutes for chicken chondrocytes and Sertoli cells, respectively. The results showed that 1,055 genes in chicken limb buds and 2,119 genes in chicken male gonads were significantly expressed according to the RNA-seq result **(**Supplementary Tables S7 and S8) and 407 and 263 genes were SOX9-regulating genes in the two tissues, respectively **(**Fig. 4D,E**)**. We then compared the mouse and chicken target genes using the ortholog database of Ensembl^45^. For chondrocytes, 296 of 349 SOX9-regulated genes in mice and 389 of 407 SOX9-regulated genes in chicken had orthologs in both species, and 36 genes were conserved targets in mouse and chicken chondrogenesis **(**Fig. 4F, Tables 1 and S9**)**. In contrast, only one gene, *AMH*, was a conserved target in mouse and chicken male gonad development **(**Fig. 4G, Tables 2 and S10**)**. Looking at each gene conserved in chondrocytes, previously reported chondrocyte SOX9 targets such as *COL2A1*, *COL9A1*, *COL9A2*, *COL27A1*, *HAPLN1*, and *ACAN* were conserved between mouse and chicken, and 11 of 36 conserved SOX9-regulated genes were annotated with the gene ontology (GO) term of skeletal system development (GO:0001501) **(**Tables 1 and S9**)**. These results indicated that SOX9-regulated genes are highly conserved in developing chondrocytes between mouse and chicken and many of them contribute to skeletal system development, whereas only a limited number of SOX9-regulated genes are conserved in the Sertoli cells between the two species.

**Table 1:** List of conserved SOX9-regulated genes in developing chondrocytes.

Conserved SOX9-regulated genes in chondrocytes (36 genes)			
Ensembl Gene ID (mouse)	Gene symbol (mouse)	Ensembl Gene ID (chicken)	Gene symbol (chicken)
ENSMUSG00000003437	Paf1	ENSGALG00000027238	PAF1
**ENSMUSG00000008090**	**Fgfrl1**	**ENSGALG00000015725**	**FGFRL1**
ENSMUSG00000019846	Lama4	ENSGALG00000015001	LAMA4
**ENSMUSG00000020583**	**Matn3**	**ENSGALG00000016478**	**MATN3**
ENSMUSG00000020644	Id2	ENSGALG00000016403	ID2
**ENSMUSG00000021613**	**Hapln1**	**ENSGALG00000015627**	**HAPLN1**
**ENSMUSG00000022483**	**Col2a1**	**ENSGALG00000013587**	**COL2A1**
ENSMUSG00000022816	Fstl1	ENSGALG00000014970	FSTL1
**ENSMUSG00000024899**	**Papss2**	**ENSGALG00000003689**	**PAPSS2**
ENSMUSG00000025555	Farp1	ENSGALG00000016886	FARP1
ENSMUSG00000025646	Atrip	ENSGALG00000004670	ATRIP
ENSMUSG00000026147	Col9a1	ENSGALG00000015970	COL9A1
ENSMUSG00000026864	Hspa5	ENSGALG00000001000	HSPA5
ENSMUSG00000027570	Col9a3	ENSGALG00000005628	COL9A3
**ENSMUSG00000027966**	**Col11a1**	**ENSGALG00000005180**	**COL11A1**
ENSMUSG00000028161	Ppp3ca	ENSGALG00000012280	PPP3CA
**ENSMUSG00000028626**	**Col9a2**	**ENSGALG00000027526**	**COL9A2**
**ENSMUSG00000030607**	**Acan**	**ENSGALG00000006725**	**ACAN**
ENSMUSG00000031546	Gins4	ENSGALG00000026581	GINS4
ENSMUSG00000031930	Wwp2	ENSGALG00000000699	WWP2
ENSMUSG00000032431	Crtap	ENSGALG00000011972	CRTAP
ENSMUSG00000032911	Cspg4	ENSGALG00000002678	CSPG4
ENSMUSG00000033565	Rbfox2	ENSGALG00000012540	RBFOX2
ENSMUSG00000033596	Rfwd3	ENSGALG00000002755	RFWD3
ENSMUSG00000034271	Jdp2	ENSGALG00000010322	JDP2
ENSMUSG00000036158	Prickle1	ENSGALG00000009556	PRICKLE1
**ENSMUSG00000040533**	**Matn1**	**ENSGALG00000000548**	**MATN1**
**ENSMUSG00000045672**	**Col27a1**	**ENSGALG00000006958**	**COL27A1**
ENSMUSG00000047409	Ctdspl	ENSGALG00000005710	CTDSPL
ENSMUSG00000053007	Creb5	ENSGALG00000011137	CREB5
ENSMUSG00000054196	Cthrc1	ENSGALG00000016071	CTHRC1
ENSMUSG00000055653	Gpc3	ENSGALG00000006087	GPC3
**ENSMUSG00000056758**	**Hmga2**	**ENSGALG00000009875**	**HMGI-C**
ENSMUSG00000056972	Magel2	ENSGALG00000028724	NDNL2
ENSMUSG00000064080	Fbln2	ENSGALG00000005120	FBLN2
ENSMUSG00000067274	Rplp0	ENSGALG00000023294	RPLP0

SOX9-regulated genes conserved between mouse and chicken in chondrocytes are shown. The genes belonging to the GO term: Skeletal system development (GO:0001501), are highlighted in bold. SOX9, SRY (sex-determining region Y)-box 9; GO, gene ontology.

**Table 2:** List of conserved SOX9-regulated genes in developing Sertoli cells.

Conserved SOX9-regulated genes in Sertoli cells (1 gene)			
Ensembl Gene ID (mouse)	Gene symbol (mouse)	Ensembl Gene ID (chicken)	Gene symbol (chicken)
*ENSMUSG00000035262*	*Amh*	*ENSGALG00000024368*	*AMH*

SOX9-regulated genes conserved between mouse and chicken in Sertoli cells are shown. The gene belonging to the GO term: developmental process involved in reproduction (GO:0003006), is highlighted in italic. SOX9, SRY (sex-determining region Y)-box 9; GO, gene ontology.

## Discussion

During embryogenesis, SOX9 is expressed in chondrocytes and Sertoli cells, playing critical roles in cartilage and testis development, not only in mammals, but also in non-mammalian vertebrates^11,12,19,46^. In the present study, we comprehensively analyzed the SOX9-dependent gene regulatory network in different cells (chondrocytes and Sertoli cells) of two vertebrates, mouse and chicken, using ChIP-seq and RNA-seq. This genome-wide analysis demonstrated cell type-specific SOX9 binding preferences in chondrocytes and Sertoli cells, and a different conservation of its regulating gene network between the two vertebrates.

Our analysis of the positional relationship between SOX9 binding regions and genes showed that characteristics were shared between mouse and chicken. In male gonads, more than half of the SOX9 binding sites were upstream of the genes near the TSS in both species, whereas SOX9 binding to “other regions” was more frequent in the limb bud than in the male gonads. Here, “other regions” means regions distant from any gene and may include enhancers. These findings were consistent with a previous report that the binding of SOX9 at a site far from the gene was predominant for cartilage-related gene targets and that binding to the upstream regions of genes closer to the TSS was dominant on non-cartilage-related target genes^23^. Our data revealed that these trends are shown not only in mice, but also in chickens.

*De novo* motif analysis revealed that the SOX palindromic motif was enriched in SOX9 binding regions in chondrocytes, but not in Sertoli cells. Previous studies also showed that the SOX palindromic motif was enriched in SOX9 binding regions in primary cultured rib chondrocytes from new born mice and in rat chondrosarcoma swarm (RCS) cells^23,24^. These observations, together with those of the present study, are consistent with previous reports that SOX9 homodimer formation and binding to a SOX palindromic motif sequence is critical for chondrocyte-specific gene expression^31,32^. The enrichment of the palindromic motif was observed in both mouse and chicken chondrocyte SOX9 binding regions, suggesting the conservation of SOX9 homodimer formation in chondrogenic gene regulation. Previous reports have shown that SOX9 regulate non-cartilage genes by binding around the TSS via associating other transcriptional factor (termed as Class I engagement) while SOX9 regulate cartilage-related genes by binding upstream enhancer element via SOX9 homodimer (termed as Class II engagement) during developmental mouse rib chondrocyte^23^. Our findings on the SOX9’s binding position and binding motif in mouse and chicken chondrocytes represent the Class II engagement and show that this regulation have been evolutionarily conserved for at least several hundred million years since the common ancestor of mammals and birds.

The present data showed that the primary SOX9 binding motif was CCAAT in male chicken gonads. In addition, in the analysis of SOX9 binding sites in mouse gonads, the CCAAT motif was listed higher than the consensus SOX motif, (A/T)(A/T)CAA(T/A)G. Furthermore, in the limb buds of both species, CCAAT was also listed, although it was ranked lower. SOX proteins do not function when they bind to DNA alone, and in addition to forming homodimers and binding to the target sites, they can form complexes with another partner^47^. Nuclear transcription factor Y subunit alpha (NFYA) is one of these partners, and SOX9-NFYA complexes commonly bind to the CCAAT sequence to exert their function^48^. Thus, our findings suggested that homodimers of SOX9 often bind and function in developing chondrocytes, whereas SOX9 proteins tend to form a complex with partners to exert their function in developing Sertoli cells. The same results has been reported in mammals^23^; however, this is the first report of these findings in chicken. In summary, in both mice and chickens, SOX9 forming homodimers at the far end of genes and binding to palindromic SOX sequences is more common in chondrocytes and SOX9 forming complexes with partners and binding closer to the TSS is common in Sertoli cells.

Our comparative analysis identified many genes associated with Gene Ontology annotation “skeletal system development” (GO:0001501) among the 36 conserved SOX9-regulated genes in developing chondrocytes, suggesting high conservation of critical chondrocyte targets of SOX9. Among these target genes, *COL2A1*^49^, *HAPLN1*^50^, *COL9A2*^51^, *COL11A1*^52^, *MATN3* (encoding matrilin 3)^53^, *PAPSS2* (encoding 3′-phosphoadenosine 5′-phosphosulfate synthase 2)^54,55^, and *FGFRL1* (encoding fibroblast growth factor receptor like 1)^56^ were previously identified as skeletal dysplasia responsible genes. In addition, *ACAN*^57^, *COL27A1*^58^, *MATN1* (encoding matrilin 1)^59^, and *HMGA2* (encoding high mobility group AT-hook 2)^60^ were reported to be involved in cartilage development or chondrocyte differentiation. In addition, *CTHRC1* (encoding collagen triple helix repeat containing 1), *WWP2* (WW domain containing E3 ubiquitin protein ligase 2) and *COL9A3* are reported to be involved in skeletal system development, although the current reference database does not assign the GO term to these genes. *CTHRC1* is crucial for bone remodeling^61^, *WWP2* is a positive regulator of SOX9 transcriptional activity during chondrogenesis^62^ and *COL9A3* is considered as a late chondrocyte-differentiation marker^63^. These genes are likely to be critical and conserved regulators of chondrogenesis as downstream targets of SOX9. Thus, while many SOX9 target genes are conserved and function in developing chondrocytes between mouse and chicken, only *AMH* was a conserved target gene in Sertoli cells. In addition to *AMH*, some genes that were annotated with GO term of developmental process involved in reproduction (GO:0003006) were listed as SOX9-regulated genes in Sertoli cells in each species, but they were not conserved. Thus, it is necessary to consider the difference of functional network of SOX9 in sex determination of both species, especially considering that their sex chromosome systems are different: XX is female and XY is male in mouse, while in chicken ZZ is male and ZW is female. In mammals, the control of *SOX9* by SRY, whose gene is located on the Y chromosome, is deeply involved in gonad development^18,44^; however, no *SRY* gene has been identified in chicken. In chicken, regulation of *SOX9* by DMRT1, whose gene is located on chromosome Z, is important in male gonad development^64^ and mouse DMRT1 is also involved in male gonad development^65^; however, its gene is present on chromosome 19, not on the sex chromosome. Thus, because of differences in the sex chromosome system, the initiation factor for sex determination is different for mouse and chicken. By contrast, both *SOX9* and *AMH*, which is involved in Müllerian duct regression and masculinization, are conserved between the two species, their expression has been confirmed in gonadal formation^66,67^ and a putative SOX binding site commonly exists upstream of the *AMH* gene^22,26^. The control of *Amh* by SOX9 in mouse is well known and *Sox9* was first detected in E10.5 and *Amh* was first detected in E11.5^68,69^. In chicken, although *AMH* expression precedes *SOX9* expression in male gonads, their expression is strongly correlated with gonadal development in terms of timing and location. *AMH* was detected at HH28 and then there was a surge in *AMH* expression at HH30 when *SOX9* expression was initiated^67^. This finding suggests *SOX9* expression strongly affects *AMH* expression although *SOX9* does not initiate *AMH* expression. In addition, even in chicken, persistence of the Müllerian duct was confirmed after short hairpin RNA-mediated knockdown of *AMH*^70^. Thus, although initiation factors of sex determination are different, SOX9-AMH axis is essential for normal reproductive formation in both species. Our comprehensive analysis also showed that the network around SOX9 in developing Sertoli cells is different between the two species; however, the SOX9-AMH axis is still conserved and supports the results of previous reports.

In summary, to examine the conservation of SOX9 regulatory function in chondrogenesis and gonad development, in which SOX9 plays a critical role, we performed ChIP-seq and RNA-seq using developing tissues or cells from mouse and chicken. The features of SOX9 binding positions and motifs in chondrocytes and Sertoli cells were similar in mouse and chicken; however, the conservation of SOX9-regulated genes was markedly different between the two types of cells. In developing chondrocytes, the palindromic SOX motif is important for the function of SOX9 in mouse and in chicken. In addition, many target genes are conserved between the two species and many of which are related to skeletal system development. However, in Sertoli cells, the palindromic SOX motif was less important than in chondrocytes in both species, and the number of conserved target genes was limited; however, the SOX9-AMH axis was conserved. Our results reconfirmed that the tissue-specific functions of SOX9 are highly conserved in limb skeletal development among mammal and non-mammal vertebrates, while few of its functions are conserved in the sex determination system. Present data must be meaningful as a resource for future researches.

## Methods

### Approval for animal experiments

All animal experiments were conducted in accordance with the Guidelines for Proper Conduct of Animal Experiments (Science Council of Japan) and approved by the Center for Experimental Animals of Tokyo Medical and Dental University and National Research Institute for Child Health and Development.

### Tissue preparation for the ChIP assay

Fetal murine gonads were isolated from E13 embryos as previously described^71^. Briefly, urogenital complex tissues were cut out from embryos and the gonads were separated from the mesonephros. The sex of the gonads was determined by the presence of testicular cords. When the gonads were isolated from chick E7 embryos, the sex was determined using a W chromosome specific genomic PCR method^72,73^. Forelimbs and hind limbs were collected from E13 mice and E7 chick embryos as developing limbs.

### ChIP

Tissues were homogenized using a BioMasher (Nippi, Tokyo, Japan) and cross-linked with 1% formaldehyde for 15 minutes at room temperature. The cells were then washed with cell lysis buffer (5 mM piperazine-1,4-bis-2-ethanesulfonic acid (PIPES) (pH 8.0), 85 mM KCl_2_ and 0.5% NP-40) containing protease inhibitors and resuspended in nuclear lysis buffer (1% sodium dodecylsulfate (SDS), 50 mM Tris-HCl (pH 8.0), and 10 mM ethylenediaminetetraacetic acid **(**EDTA)) containing protease inhibitors. Chromatin was fragmented to 100–400 bp using a Branson Sonifier® S-250D digital ultrasonic processor (Thermo Fisher Scientific, Waltham, MA, USA) before a 1/5 dilution in dilution buffer (16.7 mM Tris-HCl (pH 8.0), 167 mM NaCl, 1.2 mM EDTA, 0.01% SDS and 1.1% Triton X-100) containing protease inhibitors. The solution was then incubated with anti-SOX9 (Merck, Darmstadt, Germany) or normal rabbit IgG (Santa Cruz Biotechnology, Santa Cruz, CA, USA) antibodies bound to Dynabeads® Protein A (Thermo Fisher Scientific) overnight at 4 °C. Beads were washed twice with wash buffer (20 mM Tris-HCl (pH 8.0), 2 mM EDTA, 150 mM NaCl, 1% Triton X-100, and 0.1% SDS), wash buffer 2 (20 mM Tris-HCl (pH 8.0), 2 mM EDTA, 500 mM NaCl, 1% Triton X-100 and 0.1% SDS) and Tris-EDTA (TE). The immunoprecipitants were eluted from the beads at 65 °C in nuclear lysis buffer. Eluates were then incubated for 8 h at 65 °C for reverse-crosslinking, before the addition of 0.5 mg of protease K/mL for 2 h at 55 °C. DNA was purified using a MinElute PCR purification kit (QIAGEN, Valencia, CA, USA). The quantities of ChIP’d DNA were measured using a Qubit® 2.0 Fluorometer with Qubit® dsDNA HS Assay kit (Life Technologies, Carlsbad, CA, USA).

The anti-SOX9 antibody (AB5535, Merck) used in this study has previously been used for ChIP analyses and the binding specificity has been evaluated^74–76^. This antibody was used to recognize both the mouse and chicken proteins. Before performing deep sequencing, we used quantitative PCR to examine whether SOX9 could bind to known binding regions of the chondrocyte-specific target *Col2a1*^13^ and Sertoli cell-specific target *Amh*^26,27^ in each tissue.

### Quantitative PCR analysis of ChIP’d DNA

ChIP’d DNA, or whole cell extract (WCE) DNA as a quantitative standard, was analyzed quantitatively using real-time PCR with TaqMan Universal Master mix II with the uracil N-glycosylase (UNG) reagent and TaqMan Probes (Life Technologies) on an ABI PRISM® 7900HT thermal cycler (Life Technologies). Site-specific primers and TaqMan Probes for *Col2a1* SOX9 binding site (SOXBS) (forward 5′-GGGAGACCTCAGTCCTCCTT-3′, reverse 5′-GGAGGCTGTGCATTGTGG-3′ and TaqMan probe 5′-AGCCCCATTCATGAGAG-3′) and *Amh* SOXBS (forward 5′-GCTCAGGCCTCTGCAGTTAT-3′, reverse 5′-GGGTGGCCCTGCTTATATGT-3′, and TaqMan probe 5′-AAGGTCACCTTTGGTGTTG-3′) were used.

### ChIP-seq and data analysis

In the course of the above process, 368 μg of DNA from limb buds of 114 mouse embryos, 170 μg from male gonads of 333 mouse embryos, 110 μg from limb buds of 120 chicken embryos, and 87 μg from male gonads of 303 chicken embryos were obtained. In addition, 50.22 ng, 17.34 ng, 50.8 ng, and 19.36 ng of ChIP’d DNA were obtained, respectively.

DNA libraries for next-generation sequencing were prepared using TruSeq DNA Sample Preparation kit system (Illumina, San Diego, CA, USA) from 10 ng of ChIP’d DNA or WCE DNA, according to the manufacturer’s instructions, and sequenced on the HiSeq. 2000 system (Illumina).

Among the acquired 100 base paired-end sequence data, only unique sequences on the genome were mapped to the mouse (mm10) and chicken (galGal4) genomes utilizing bowtie software^77^ (http://bowtie-bio.sourceforge.net/index.shtml) version 1.0.1. The mapped SAM format files were converted to BAM files using samtools^78^ (http://samtools.sourceforge.net/) version 0.1.19, and ChIP’d DNA enriched regions were detected using the MACS software^79^ (http://liulab.dfci.harvard.edu/MACS/) version 1.4.2 with a p-value < 1 e-7 and a false discovery rate (FDR) <1%. To evaluate the ChIP-seq peak calling among the experiments, we depicted histograms for the distribution of fold enrichment value of the peaks **(**Supplementary Fig. S7), using R version 3.5.3^80^ Our histograms showed that fold enrichment value for ChIP-seq peaks shared a tendency to accumulate in the bin ranges of 10–15 and 15–20 for both limb bud and male gonad samples. We also quantified skewness and kurtosis by using the R package “moments”^81^. We found that these values were similar for limb and gonad samples. These data suggest that the quality of our ChIP-seq peak data were comparable enough between limb bud and male gonad samples. To visualize the ChIP-seq data, RPKM (reads per kilobase of transcript per million mapped reads) normalization and bigwig conversion were conducted using deepTools version 3.1.3 software^82^. The data were visualized using Integrative Genomics Viewer (IGV)^83^. Detailed information about the sequencing course is included in Supplementary Table S11.

The genomic position of the ChIP-seq peaks were separated into seven regions based on ensembl gene information: 5′-UTR, 3′-UTR, exon, intron, 10 kb upstream regions from the TSS of genes, 10 kb downstream regions from the end of the 3′-UTR, and others. This analysis was conducted with two different data sets, one data set includes all significant peaks detected the other data set includes top 1000 peaks. The resultant patterns were equivalent in the two analyses, which is a proof for the robustness of the analysis. Only the results using the former data set are described in this manuscript.

### *De novo* motif discovery, motif frequency analysis, and binding site conservation analysis

Genome sequence ±50 bp from the summit of the SOX9 ChIP-seq peaks were analyzed using MEME-ChIP (http://meme.ebi.edu.au/meme/index.html)^29^ to obtain enriched motifs. MEME-ChIP analysis was performed by setting options to detect sequences of 18 bases or more and using default setting for the generation of background sequence. Detailed MEME-ChIP settings are as follows. (MEME-ChIP normal setting: -db JASPAR2018_CORE_vertebrates_redundant_pfms.meme -meme-nmotifs 10 -meme-maxsize 1000000 -meme-minw 18 -meme-p 4)(MEME-ChIP palindrome setting: -db JASPAR2018_CORE_vertebrates_redundant_pfms.meme -meme-nmotifs 10 -meme-maxsize 1000000 -meme-minw 18 -meme-p 4 -meme-pal). JASPAR2018_CORE_vertebrates_redundant_pfms.meme was downloaded from http://jaspar.genereg.net. To identify the indicated motifs in the ChIP-seq peak regions, individual motifs were scanned on the plus and minus strands of the regions using profit in EMBOSS^33^ (http://emboss.bioinformatics.nl/cgi-bin/emboss/profit). The position weight matrices corresponding to the MEME-ChIP output data (Red line and character in Figs 2B and 3B**)** were created by EMBOSS prophecy (http://emboss.bioinformatics.nl/cgi-bin/emboss/prophecy) and the frequency of the indicated motifs in the ChIP-seq peak regions were scanned by profit in EMBOSS with a relative profile score threshold of >75%. The conservation of genome sequence ±100 bp from the summit of the SOX9 ChIP-seq peaks was evaluated using the phastCons score generated from 60 vertebrate genomes (Siepel *et al*. 2005). The phastCons score is a value between 0 and 1. The average phastCons scores of SOX9 ChIP-seq peaks were compared using the Mann–Whitney U test.

### Cell preparation and RNA extraction for RNA sequencing

E13 mouse chondrocytes in limb buds: We crossed *Col2a1*/Cre transgenic mice^84^ and R26R (Rosa 26 reporter) mice^85^ to obtain embryos expressing lacZ in their chondrocytes. E13 embryo limb buds were dissected out, minced, and then treated with 3 mg/mL collagenase at 37 °C for 60 minutes. After pipetting, the samples were passed through a 100-µm cell strainer and collected as separate cells. Subsequently, the cells were treated with fluorescein di-β-D-galactopyranoside (FDG) using FluoReporter lacZ Flow Cytometry Kits (Molecular Probes, Leiden, Netherlands) to obtain E13 mouse limb bud chondrocytes, which could be collected using fluorescence activated cell sorting (FACS). FACS was performed using MoFlo XDP (Beckman Coulter, Fullerton, CA, USA). From the recovered cells, RNA was extracted using a ReliaPrep RNA Cell Miniprep System (Promega, Madison, WI, USA).

E13 mouse Sertoli cells in male gonads: We obtained Sertoli cells by collecting male gonads from E13 embryos of Sry-GFP mice^86^, treating them with collagenase, passing them through a cell strainer, and sorting Sertoli cells using FACS. From these cells, RNA samples were obtained using the ReliaPrep RNA Cell Miniprep System (Promega).

E7 chicken chondrocytes and Sertoli cells: Unlike mice, it was difficult to extract these cells from chickens using transgenic animals. Therefore, RNA extracted from E7 chicken limb buds and male gonads were used as substitutes for chondrocyte and Sertoli cell RNA, respectively. Each tissue was taken from the ChIP experiment, homogenized using the BioMassher, and the RNA was extracted from them using the ReliaPrep RNA Tissue Miniprep System (Promega).

E13 mouse and E7 chicken whole embryos: As controls, whole embryos were obtained at the same developmental stage. They were homogenized using the BioMassher, and RNA was extracted from them using the ReliaPrep RNA Tissue Miniprep System (Promega).

### RNA sequencing

We chose Quartz-seq^38^ for RNA sequencing in the present experimental system, because the obtained numbers of E13 mouse chondrocytes and Sertoli cells were small: 723; 1,040; and 745 cells (n = 3) were obtained as E13 mouse chondrocyte samples and 914; 1,091; and 1,291 cells (n = 3) were obtained as E13 mouse Sertoli cell samples. Library preparation of all RNA samples followed the procedure of Quartz-seq, as previously described^38^. The generated samples were sequenced using an Illumina Hiseq. 1500 instrument. Sequence reads were mapped to the reference genomes (mm10 and galGal4) using Tophat version 2.1.1 software^87^. Mapped reads were then assembled into complete transcripts using Cufflinks version 2.1.1. software^88^. Detailed information about the sequencing results is included in Supplementary Table S12. PCA plots were generated using bamCoverage, multibigwigSummary and plotPCA from the deepTools version 3.1.3 software^82^. bamCoverage calculated the normalized sequence read coverage using optional parameter–normalizeUsingRPKM, multibigwigSummary summarized each sequence libraries using an optional parameter–binSize 10000. plotPCA was used to visualize the PCA plot with an optional parameter–log2. To visualize the RNA-seq data, RPKM (reads per kilobase of transcript per million mapped reads) normalization and bigwig conversion was conducted using deepTools version 3.1.3 software^82^. The data was visualized using Integrative Genomics Viewer (IGV)^83^.

### Target gene prediction and conservation analysis

Putative SOX9 target genes were identified if the ChIP-seq peak was on its cis-regulatory region, defined from 10 kb upstream from the TSS to the 3′-UTR. Gene and genome position information were retrieved from the UCSC Table Browser^89^ (http://genome.ucsc.edu). Among these gene groups, highly expressed genes in each cell were regarded as SOX9-regulated genes in the present study. We defined highly expressed genes as those whose FPKM was more than 1.0, was 1.5 times higher than in whole embryo, and their q-values of the comparisons were less than 0.05.

The SOX9 target genes among mouse and chicken were compared after matching orthologs using an ortholog database (Ensembl version 85 in Ensembl BioMart)^45^. Among the genes listed in each species, only genes present in both mouse and chicken were compared. All Venn diagrams were drawn using BioVenn^90^.

The database of GO terms was obtained from GO enrichment analysis (http://geneontology.org/)^91,92^. All mouse and chicken genes were converted into human genes using an ortholog database in Ensembl version 85^45^ and annotated to human GO terms. The annotation version was GO Ontology Database Released 2018-02-02.

### Sequencing data

The raw sequencing data from this study have been submitted to the DNA Data Bank Japan (DDBJ; http://www.ddbj.nig.ac.jp) under the accession numbers DRA003255, DRA003256 and DRA007771.

## Supplementary information

Supplementary Figures and Legends

Supplementary Tables

## Data Availability

Referential genome sequences mm10 and galGal4 were available in (http://hgdownload.cse.ucsc.edu/goldenPath/mm10/bigZips/mm10.2bit) and (http://hgdownload.cse.ucsc.edu/goldenPath/galGal4/bigZips/galGal4.2bit). The following software was used in analysis of next generation sequencing bowtie software version 1.0.1 (http://bowtie-bio.sourceforge.net/index.shtml) samtools version 0.1.19 (http://samtools.sourceforge.net/). MACS software version 1.4.2 (http://liulab.dfci.harvard.edu/MACS/) deepTools version 3.1.3 (https://deeptools.readthedocs.io/en/develop/). IGV version 2.3.72 (http://software.broadinstitute.org/software/igv/). MEME suite 4.11.2 (http://meme-suite.org/index.html). EMBOSS version 6.6.0 (http://emboss.bioinformatics.nl/cgi-bin/emboss/profit). Tophat version 2.1.1 (https://ccb.jhu.edu/software/tophat/index.shtml). Cufflinks version 2.1.1 (http://cole-trapnell-lab.github.io/cufflinks/). Ensembl version 85 (http://jul2016.archive.ensembl.org/biomart/martview/) and Gene Ontology Database released 2018-02-02 (http://www.geneontology.org/) were used for analysis of SOX9 target genes.
