# Supplementary material for: Comparative analysis demonstrates cell type-specific conservation of SOX9 targets between mouse and chicken
Source: Sci Rep. 2019 Aug 29;9:12560. doi: 10.1038/s41598-019-48979-4 (PMC6715657; doi:10.1038/s41598-019-48979-4)
Supplement: Supplementary file 1 — Supplementary Figures and Legends [file 41598_2019_48979_MOESM1_ESM.pdf]

Title: Comparative analysis demonstrates cell type-specific conservation of SOX9 targets between mouse and chicken

Authors: Satoshi Yamashita[1,2,†], Kensuke Kataoka[1,3,†], Hiroto Yamamoto[1,4,†], Tomoko Kato[2], Satoshi Hara[2], Katsushi Yamaguchi[5], Claire Renard-Guillet[6], Yuki Katou[6], Katsuhiko Shirahige[6], Haruki Ochi[7], Hajime Ogino[8], Tokujiro Uchida[4] , Masafumi Inui[2,9], Shuji Takada[2], Shuji Shigenobu[5,*], Hiroshi Asahara[1,10,11,*]

Affiliations:
[1]Department of Systems BioMedicine, Tokyo Medical and Dental University, 1-5-45 Yushima, Bunkyo-ku, Tokyo 113-8510, Japan
[2]Department of Systems BioMedicine, National Institute for Child Health and Development, 2-10-1 Okura, Setagaya-ku, Tokyo, 157-8535, Japan
[3]Research Fellow of Japan Society for the Promotion of Science, Tokyo, Japan.
[4]Department of Anesthesiology, Tokyo Medical and Dental University, Graduate School of Medical and Dental Sciences, 1-5-45 Yushima, Bunkyo-ku, Tokyo 113-8510, Japan
[5]Functional Genomics Facility, National Institute for Basic Biology, 38, Nishigonaka Myodaiji Okazaki Aichi 444-8585, Japan
[6]Laboratory of Genome Structure and Function Center for Epigenetic Disease, Institute of Molecular and Cellular Biosciences, The University of Tokyo, 1-1-1 Yayoi, Bunkyo-ku, Tokyo, 113-0032, Japan
[7]Institute for Promotion of Medical Science Research, Faculty of Medicine, Yamagata University, 2-2-2 Iida-nishi, Yamagata, 990-9585, Japan
[8]Amphibian Research Center, Hiroshima University, 1-3-1 Kagami-yama, Higashi-Hiroshima, Hiroshima, 739-8526, Japan
[9]Laboratory of Animal Regeneration Systemology, Department of Life Sciences, School of Agriculture, Meiji University, 1-1-1 Higashi-Mita Tama-ku Kawasaki Kanagawa 214-8571, Japan
[10]Department of Molecular Medicine, The Scripps Research Institute, California, 92037, USA.
[11]AMED-CREST, Japan Agency for Medical Research and Development (AMED), Tokyo, Japan

†These authors contributed equally to this work.
*To whom correspondence should be addressed:
Hiroshi Asahara
Tel: +81 3 5803 5015
Email: asahara@scripps.edu and asahara.syst@tmd.ac.jp
Shuji Shigenobu
Tel: +81 564 55 7670
Email: shige@nibb.ac.jp

Figure S1.

MEME-ChIP normal setting

Logo	E-value
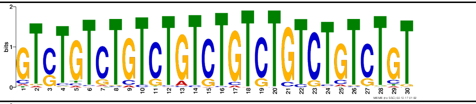	7.4e-885
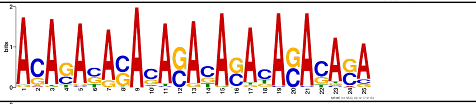	2.4e-177
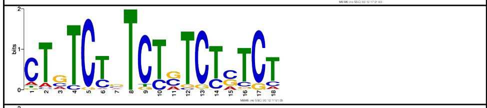	4.4e-72
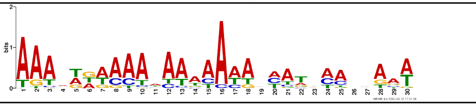	3.9e-28
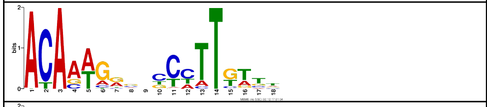	6.5e-25
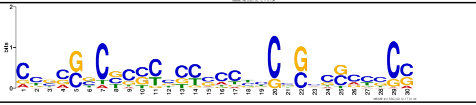	1.0e-10

MEME-ChIP palindrome setting

Logo	E-value
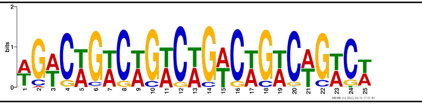	1.3e-437
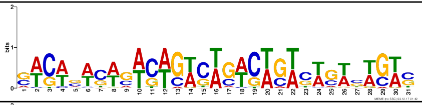	9.3e-64
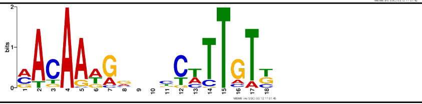	1.2e-14
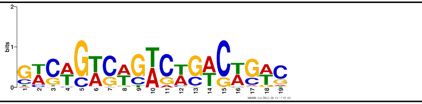	9.1e-4

DREME

Logo	E-value	Logo	E-value	Logo	E-value
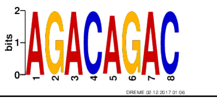	7.6e-117	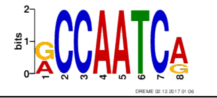	4.8e-17	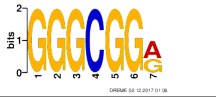	9.3e-6
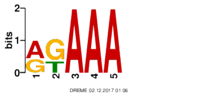	1.6e-61	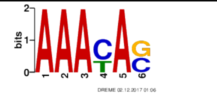	2.3e-10	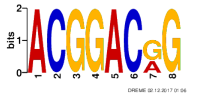	8.1e-5
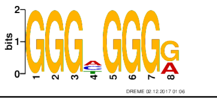	2.2e-33	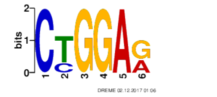	3.4e-9	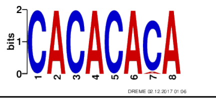	7.2e-4
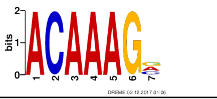	1.8e-25	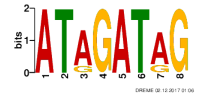	1.9e-8	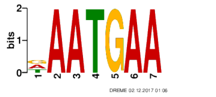	3.0e-3
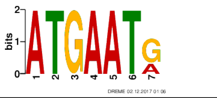	2.7e-22	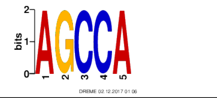	4.1e-7	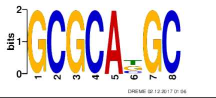	6.3e-3
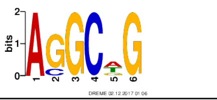	4.2e-20	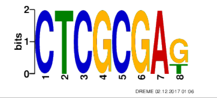	4.2e-6	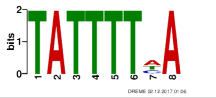	1.4e-2

Figure S2.

MEME-ChIP normal setting

**Logo**	**E-value**
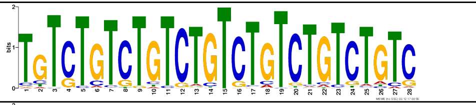	4.3e-648
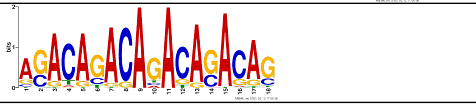	1.6e-96
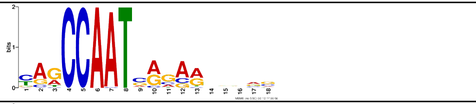	2.2e-71
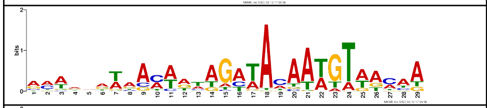	9.9e-38
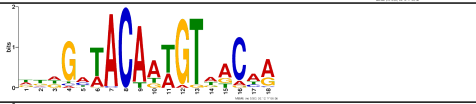	2.4e-22
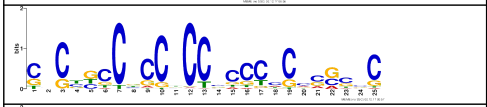	9.9e-16
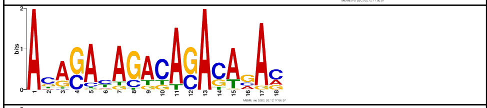	1.0e-11
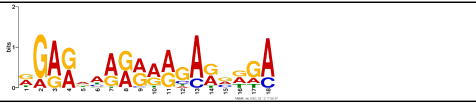	7.3e-11
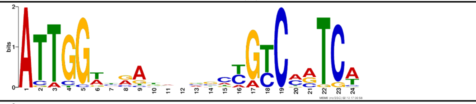	1.4e-2
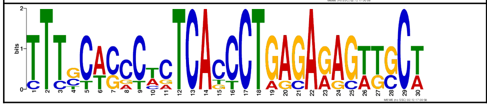	2.9e-2

MEME-ChIP palindrome setting

**Logo**	**E-value**
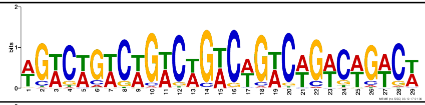	9.6e-359
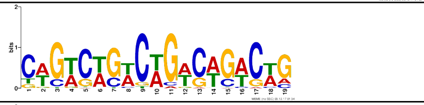	1.7e-26
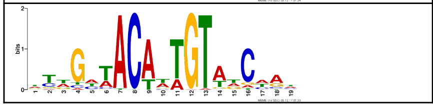	9.7e-25

DREME

**Logo**	**E-value**
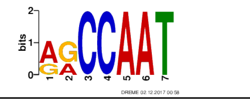	1.6e-53
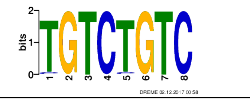	3.6e-27
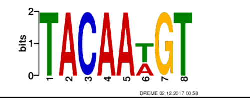	7.5e-10
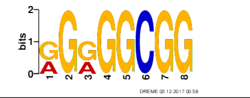	6.3e-9
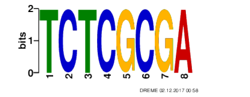	5.7e-4
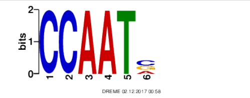	1.4e-3
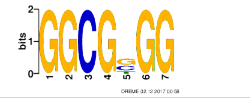	1.9e-2

Figure S3.

MEME-ChIP normal setting

Logo	E-value
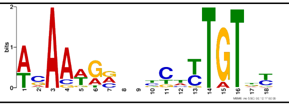	3.7e-83
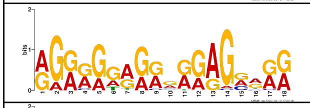	3.7e-34
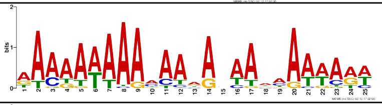	1.4e-6
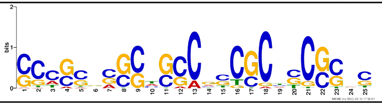	4.3e-6

MEME-ChIP palindrome setting

Logo	E-value
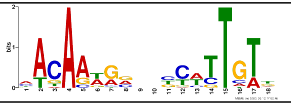	1.0e-64

DREME

Logo	E-value	Logo	E-value	Logo	E-value
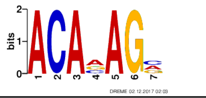	1.8e-290	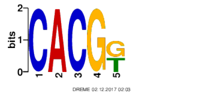	2.0e-25	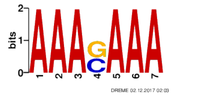	1.5e-5
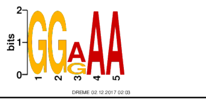	4.1e-238	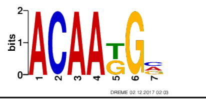	4.3e-18	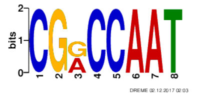	3.6e-5
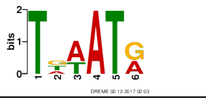	7.4e-187	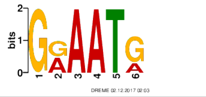	2.0e-17	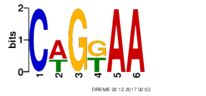	2.5e-4
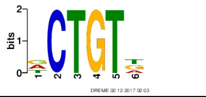	4.1e-129	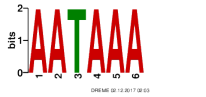	9.3e-14	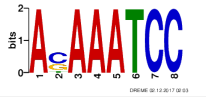	1.0e-3
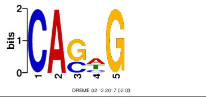	2.2e-50	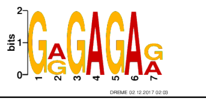	1.2e-12	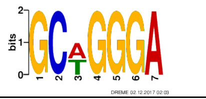	5.1e-3
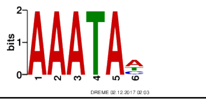	2.8e-47	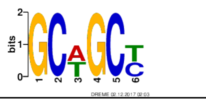	1.6e-12	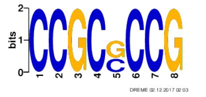	2.6e-2
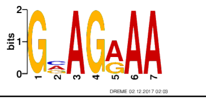	1.8e-38	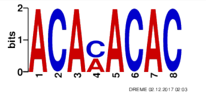	5.0e-10	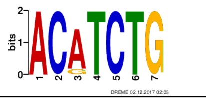	2.7e-2
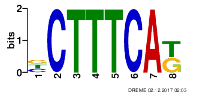	1.7e-28	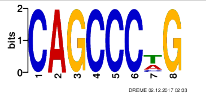	9.3e-7		
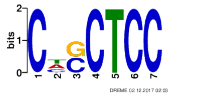	7.5e-28	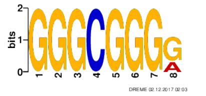	3.3e-6		

Figure S4.

MEME-ChIP normal setting

Logo	E-value
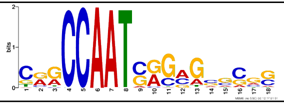	6.1e-325
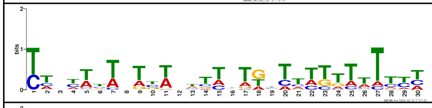	2.1e-128
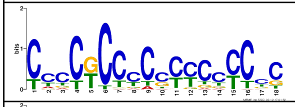	2.8e-60
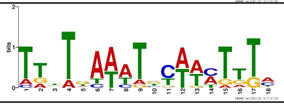	2.5e-7
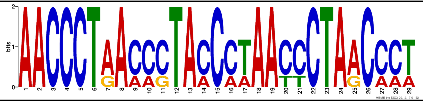	8.8e-3

MEME-ChIP palindrome setting

Logo	E-value
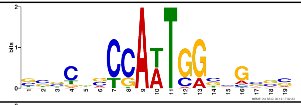	1.7e-113
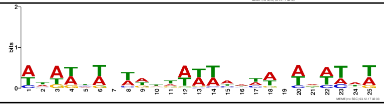	1.0e-56

DREME

Logo	E-value	Logo	E-value	Logo	E-value
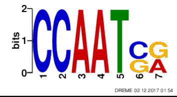	3.1e-113	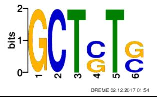	3.9e-6	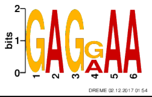	7.8e-3
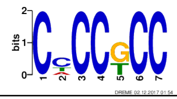	1.7e-22	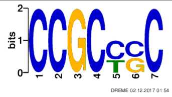	7.7e-6	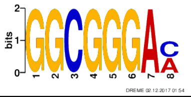	9.5e-3
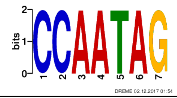	1.4e-18	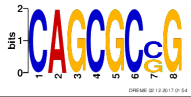	1.9e-4		
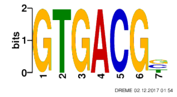	1.7e-9	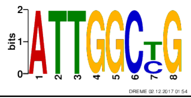	2.9e-4		

Figure S5.

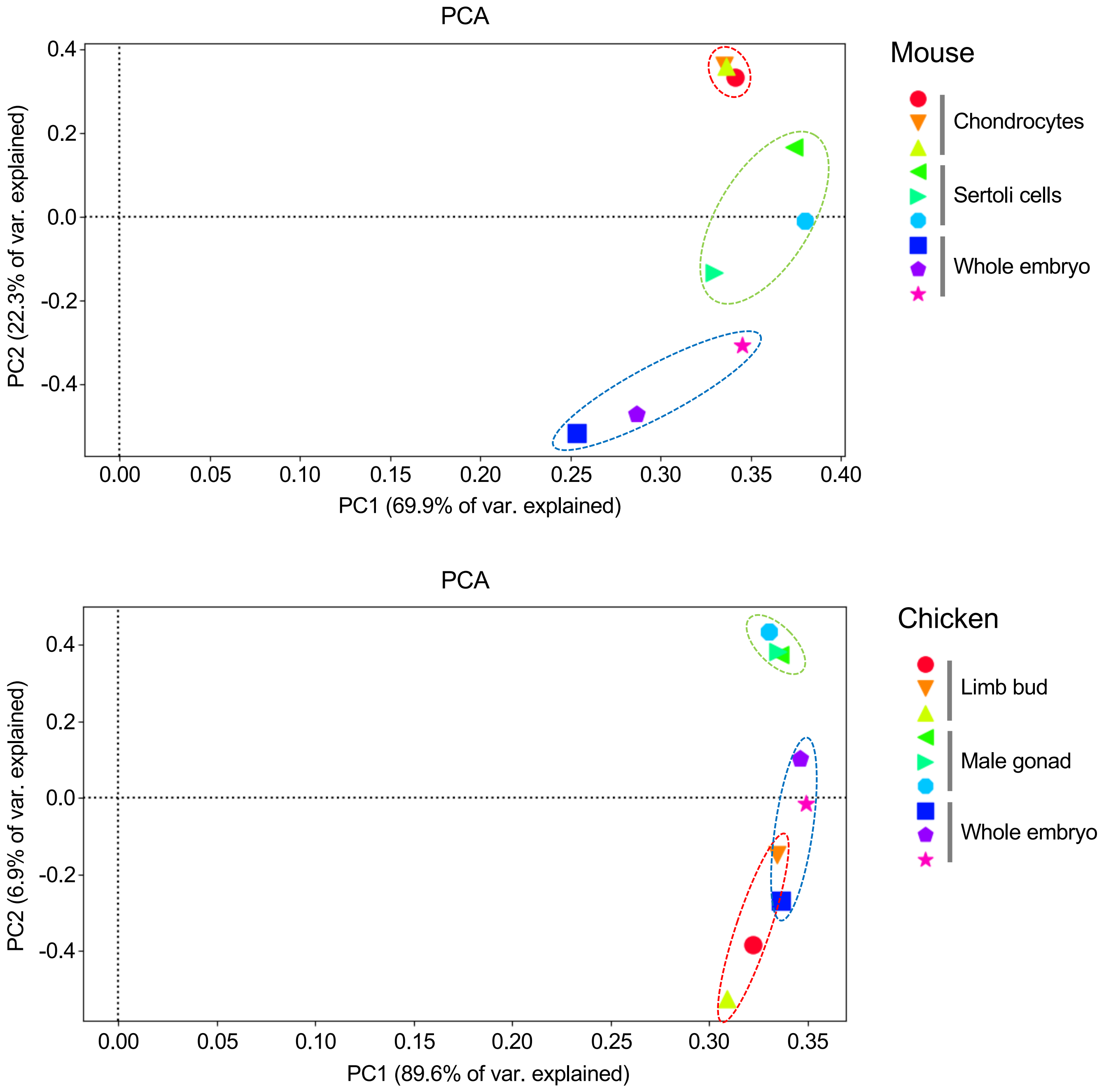
PCA
0.4
0.2
0.0
-0.2
-0.4
PC2 (22.3% of var. explained)
0.00
0.05
0.10
0.15
0.20
0.25
0.30
0.35
0.40
PC1 (69.9% of var. explained)
Mouse
Chondrocytes
Sertoli cells
Whole embryo
PCA
0.4
0.2
0.0
-0.2
-0.4
PC2 (6.9% of var. explained)
0.00
0.05
0.10
0.15
0.20
0.25
0.30
0.35
PC1 (89.6% of var. explained)
Chicken
Limb bud
Male gonad
Whole embryo

Figure S6.

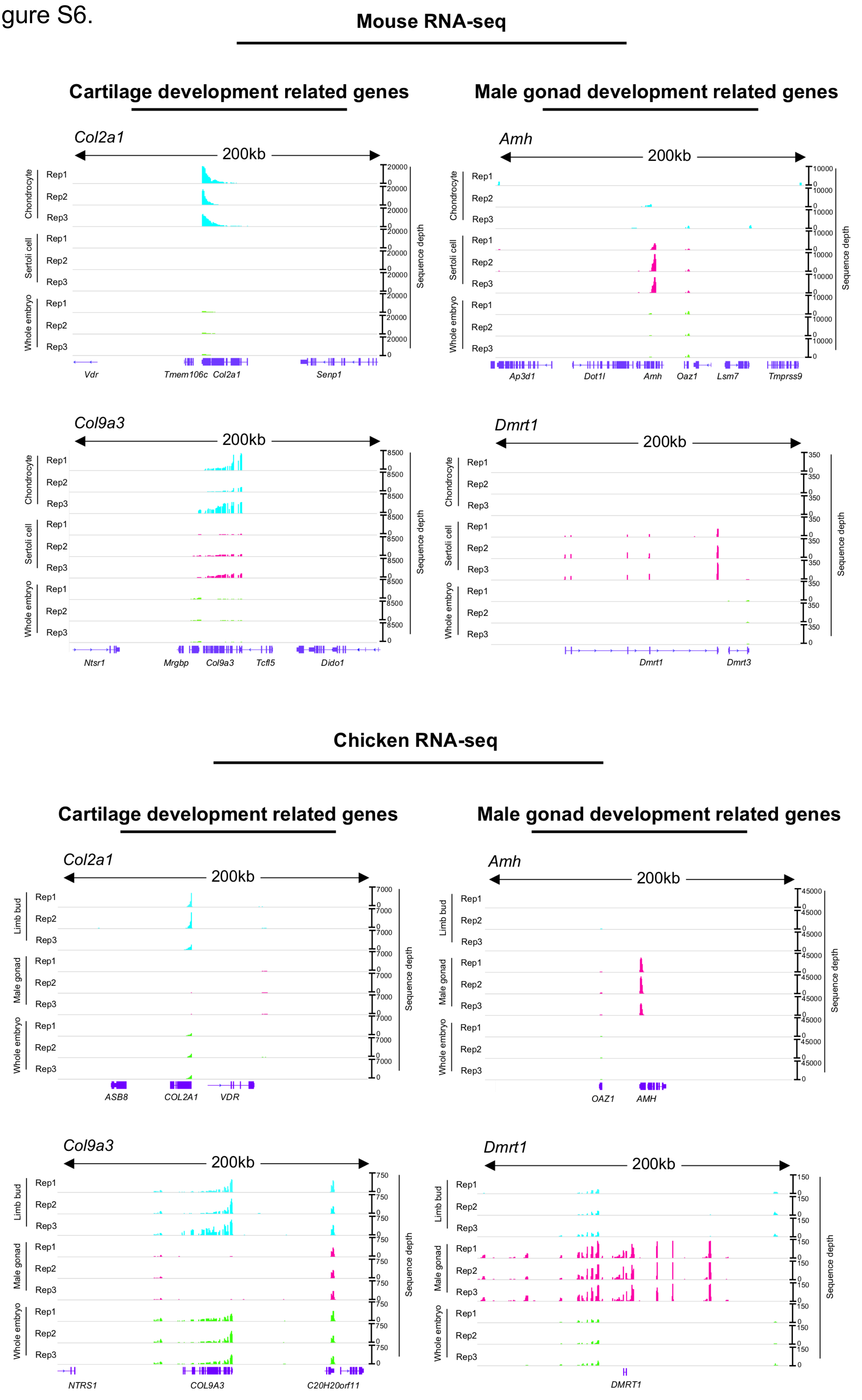
Mouse RNA-seq
Cartilage development related genes
Male gonad development related genes
Col2a1
200kb
Chondrocyte
Sertoli cell
Whole embryo
Rep1
Rep2
Rep3
Sequence depth
20000
0
Vdr
Tmem106c Col2a1
Senp1
Amh
10000
Ap3d1
Dot1l
Amh
Oaz1
Lsm7
Tmprss9
Col9a3
8500
Ntsr1
Mrgbp
Col9a3
Tcfl5
Dido1
Dmrt1
350
Dmrt1
Dmrt3
Chicken RNA-seq
Cartilage development related genes
Male gonad development related genes
Col2a1
Limb bud
Male gonad
Whole embryo
7000
ASB8
COL2A1
VDR
Amh
45000
OAZ1
AMH
Col9a3
750
NTRS1
COL9A3
C20H20orf11
Dmrt1
150
DMRT1

Figure S7.

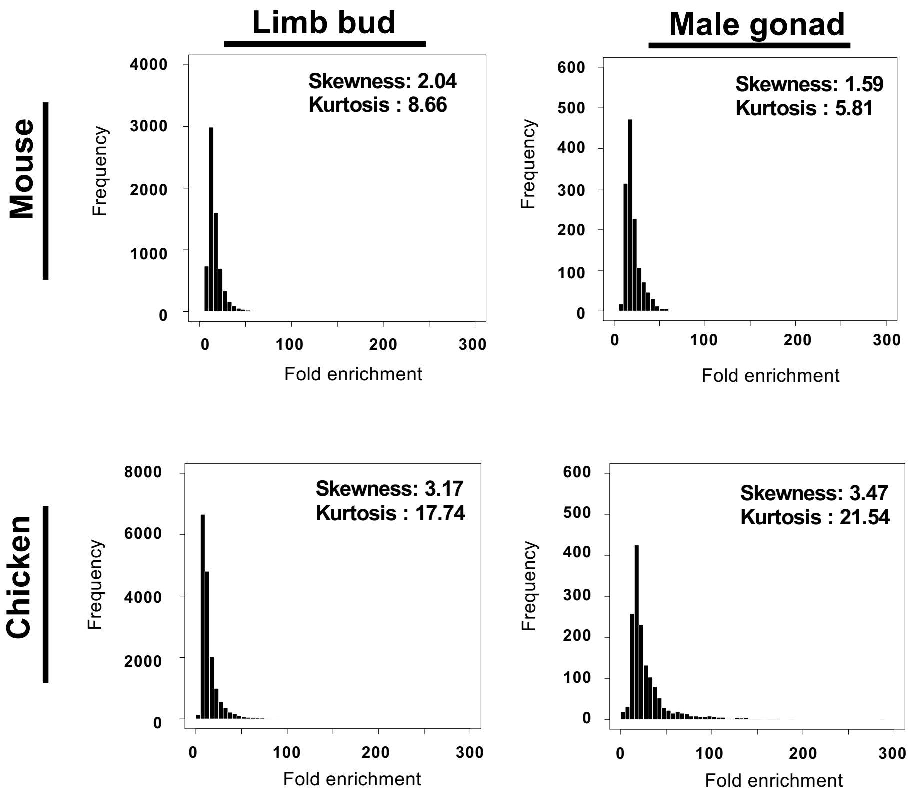
Limb bud
Male gonad
Mouse
Chicken
Skewness: 2.04
Kurtosis : 8.66
Skewness: 1.59
Kurtosis : 5.81
Skewness: 3.17
Kurtosis : 17.74
Skewness: 3.47
Kurtosis : 21.54
Frequency
Fold enrichment
0
100
200
300
1000
2000
3000
4000
400
500
600
4000
6000
8000

Supplementary Figure Legends.

Supplementary Figure S1. All motifs of SOX9 chromatin immunoprecipitation sequencing (ChIP-seq) peak regions in embryonic day (E)13 mouse limb buds. All motifs with an E-value < 0.05 in our MEME-ChIP analysis are shown. Red-highlighted E-values show that the corresponding motifs include sequences similar to single or palindromic SOX motifs. SOX9, SRY (sex-determining region Y)-box 9.

Supplementary Figure S2. All motifs of SOX9 chromatin immunoprecipitation sequencing (ChIP-seq) peak regions in embryonic day (E)13 mouse male gonads. All motifs with an E-value < 0.05 in our MEME-ChIP analysis are shown. Red-highlighted E-values show that the corresponding motifs include sequences similar to single or palindromic SOX motifs. SOX9, SRY (sex-determining region Y)-box 9.

Supplementary Figure S3. All motifs of SOX9 ChIP-seq peak regions in embryonic day (E)7 chicken limb buds. All motifs with an E-value < 0.05 in our MEME-ChIP analysis are shown. Red-highlighted E-values show that the corresponding motifs include sequences similar to single or palindromic SOX motifs. SOX9, SRY (sex-determining region Y)-box 9.

Supplementary Figure S4. All motifs of SOX9 chromatin immunoprecipitation sequencing (ChIP-seq) peak regions in embryonic day (E)7 chicken male gonad. All motifs with an E-value < 0.05 in our MEME-ChIP analysis are shown. Red-highlighted E-values show that the corresponding motifs include sequences similar to single or palindromic SOX motifs. SOX9, SRY (sex-determining region Y)-box 9.

Supplementary Figure S5. PCA (principal component analysis) plots of the RNA-seq data. PCA plots from each triplicate sample (chondrocytes, Sertoli cells, and whole embryos of mice, and limb buds, male gonads and whole embryos of chicken) are shown with the indicated marks.

Supplementary Figure S6. Visualizing RPKM-normalized RNA-seq read-tags. Sequence tag accumulation of RNA-seq of each replicate around *COL2A1*, *COL9A3*, *AMH* and *DMRT1* are shown. Rep, Replicate; COL2A1, collagen type II alpha 1 chain; COL9A3, collagen type IX alpha 3 chain; AMH, anti-Müllerian hormone; DMRT1, doublesex and mab-3 related transcription factor

1.

Supplementary Figure S7. Histograms of ChIP-seq peak validation between tissues. MACS generated fold enrichment value for ChIP-seq peaks were depicted as histogram (bin width = 5). Distribution tendency of the fold enrichment value for ChIP-seq peaks were calculated as skewness and kurtosis value.

Supplementary Table S1. SOX9 chromatin immunoprecipitation sequencing (ChIP-seq) peak regions in embryonic day (E)13 mouse limb buds and putative target genes. 6,728 regions were detected as SOX9 ChIP-seq peak regions. Putative target genes were determined by analyzing the binding of SOX9 to cis-regulatory regions defined 10 kb upstream from the TSS to the 3′ UTR of each gene. SOX9, SRY (sex-determining region Y)-box 9; TSS, transcription start site; UTR, untranslated region.

Supplementary Table S2. SOX9 chromatin immunoprecipitation sequencing (ChIP-seq) peak regions in embryonic day (E)13 mouse male gonads and putative target genes. 1,308 regions were detected as SOX9 ChIP-seq peak regions. Putative target genes were determined by analyzing the binding of SOX9 to cis-regulatory regions defined 10 kb upstream from the TSS to the 3′ UTR of each gene. SOX9, SRY (sex-determining region Y)-box 9; TSS, transcription start site; UTR, untranslated region.

Supplementary Table S3. SOX9 chromatin immunoprecipitation sequencing (ChIP-seq) peak regions in embryonic day (E)7 chicken limb buds and putative target genes. 16,297 regions were detected as SOX9 ChIP-seq peak regions. Putative target genes were determined by analyzing the binding of SOX9 to cis-regulatory regions defined 10 kb upstream from the TSS to the 3′ UTR of each gene. SOX9, SRY (sex-determining region Y)-box 9; TSS, transcription start site; UTR, untranslated region.

Supplementary Table S4. SOX9 chromatin immunoprecipitation sequencing (ChIP-seq) peak regions in embryonic day (E)7 chicken male gonads and putative target genes. 1,516 regions were detected as SOX9 ChIP-seq peak regions. Putative target genes were determined by analyzing the binding of SOX9 to cis-regulatory regions defined 10 kb upstream from the TSS to the 3′ UTR of each gene. SOX9, SRY (sex-determining region Y)-box 9; TSS, transcription start

site; UTR, untranslated region.

Supplementary Table S5. Highly expressed genes in embryonic day (E)13 mouse chondrocytes. A highly expressed gene was defined when its fragment per kilobase of transcript per million fragments sequenced (FPKM) value in chondrocytes > 1.0, was 1.5 times higher than in the whole embryo, and the q-value of the comparison was < 0.05. 1,366 genes were found according to this definition. Ensembl gene ID, gene name, average FPKM in chondrocyte (N = 3) and in the whole embryo (N = 3), the expression ratio (fold-change) of highly expressed genes, and q-values are shown.

Supplementary Table S6. Highly expressed genes in embryonic day (E)13 mouse Sertoli cells. A highly expressed gene was defined when its fragment per kilobase of transcript per million fragments sequenced (FPKM) value in Sertoli cells > 1.0, was 1.5 times higher than in the whole embryo, and the q-value of the comparison was < 0.05. 329 genes were found according to this definition. Ensembl gene ID, gene name, average FPKM in Sertoli cells (N = 3) and in the whole embryo (N = 3), the expression ratio (fold-change) of highly expressed genes, and q-values are shown.

Supplementary Table S7. Highly expressed genes in embryonic day (E)7 chicken limb buds. A highly expressed gene was defined when its fragment per kilobase of transcript per million fragments sequenced (FPKM) value in limb buds > 1.0, was 1.5 times higher than in the whole embryo, and the q-value of the comparison was < 0.05. 1,055 genes were found according to this definition. Ensembl gene ID, gene name, average FPKM in limb buds (N = 3) and in the whole embryo (N = 3), the expression ratio (fold change) of highly expressed genes, and q-value are shown.

Supplementary Table S8. Highly expressed genes in embryonic day (E)7 chicken male gonads. A highly expressed gene was defined when its fragment per kilobase of transcript per million fragments sequenced (FPKM) value in chondrocytes > 1.0, was 1.5 times higher than in the whole embryo, and the q-value of the comparison was < 0.05. 2,119 genes were found according to this definition. Ensembl gene ID, gene name, average FPKM in male gonads (N = 3) and in the whole embryo (N = 3), the expression ratio (fold change) of highly expressed genes, and q-values are shown.

Supplementary Table S9. SOX9-regulated genes in chondrocytes. Conserved and non-conserved SOX9-regulated genes between mouse and chicken in developing chondrocytes are shown. The genes belonging to the gene ontology term of skeletal system development (GO:0001501) are highlighted in red. SOX9, SRY (sex-determining region Y)-box 9.

Supplementary Table S10. SOX9-regulated genes in Sertoli cells. Conserved and non-conserved SOX9-regulated genes between mouse and chicken in developing Sertoli cells are shown. The genes belonging to the gene ontology term of developmental process involved in reproduction (GO:0003006) are highlighted in blue. SOX9, SRY (sex-determining region Y)-box 9.

Supplementary Table S11. Sequence information obtained in the chromatin immunoprecipitation sequencing (ChIP-seq) experiments. Read numbers and aligned read numbers of each sample are shown.

Supplementary Table S12. Sequence information obtained in the RNA sequencing experiments. Read numbers and aligned read numbers of each sample are shown.
